# Cognitive Profiles and Determinants of Eligibility for Awake Surgery in Non‐Dominant Hemisphere Gliomas: A Narrative Review

**DOI:** 10.1002/brb3.70604

**Published:** 2025-05-30

**Authors:** Alice Tomaselli, Antonina Luca, Gianluca Ferini, Giuseppe Emmanuele Umana, Bipin Chaurasia, Gianluca Scalia

**Affiliations:** ^1^ Department of Medicine and Surgery Kore University of Enna Enna Italy; ^2^ REM Radioterapia srl Viagrande Italy; ^3^ Department of Neurosurgery Gamma Knife and Trauma Center, Cannizzaro Hospital Catania Italy; ^4^ Department of Neurosurgery Bhawani Hospital and Research Centre Birguni Nepal; ^5^ Neurosurgery Unit, Department of Head and Neck Surgery Garibaldi Hospital Catania Italy

**Keywords:** awake, brain mapping, cognition, glioma, hemisphere, non‐dominant

## Abstract

**Background:**

Awake surgery has become a crucial approach in glioma treatment, primarily aimed at maximizing tumor resection while preserving neurological functions. While its application to the dominant hemisphere has been well established, its use in the non‐dominant hemisphere remains underexplored. The non‐dominant hemisphere plays essential roles in visuospatial processing, social cognition, and executive functions, which can significantly impact a patient's quality of life. Despite increasing evidence of these functions, standardized protocols for intraoperative brain mapping (ioBM) in the non‐dominant hemisphere are lacking.

**Methods:**

A systematic search of the PubMed database was conducted to identify studies published between 2015 and 2024 that examined cognitive outcomes and ioBM paradigms in awake surgery for right non‐dominant hemisphere gliomas. The review included studies that assessed neuropsychological outcomes, tumor characteristics, and the extent of surgical resection. Exclusion criteria included case reports, reviews, and studies focused exclusively on dominant hemisphere gliomas. A total of 13 studies met the inclusion criteria.

**Results:**

The review identified key cognitive functions assessed during awake surgery, including speech/motor language, visuospatial cognition, executive functions, social cognition, working memory, and sensorimotor functions. Intraoperative neuropsychological assessment primarily used cortical and subcortical stimulation, with a variety of cognitive tests applied to different domains. Studies reported that direct electrical stimulation (DES) revealed functional roles for the right hemisphere in visuospatial attention, social cognition, and executive functions. Patients who underwent awake surgery demonstrated better long‐term cognitive outcomes and extended tumor resection compared to those under general anesthesia. However, variability in assessment tools and inconsistent reporting of postoperative outcomes were noted.

**Conclusion:**

Awake surgery combined with ioBM appears to be a viable approach for optimizing tumor resection while preserving cognitive functions in the non‐dominant hemisphere. However, the lack of standardized cognitive assessment protocols remains a significant challenge. Future research should focus on establishing a unified set of cognitive tests for intraoperative assessment, conducting longitudinal studies on cognitive recovery, and integrating advanced neuroimaging techniques to refine surgical mapping. Standardizing intraoperative cognitive evaluations will be essential to improving patient outcomes and expanding the application of awake surgery for non‐dominant hemisphere gliomas.

## Graphical abstract

Awake surgery with intraoperative brain mapping enables safe tumor resection in the non‐dominant hemisphere by preserving visuospatial, executive, and social cognitive functions. This review synthesizes current evidence on neuropsychological domains assessed and highlights the functional relevance of the right hemisphere. Standardized cognitive protocols are urgently needed to improve surgical outcomes and patient quality of life.

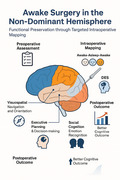

## 1 Introduction

The emergence of awake surgery as a key approach in the management of gliomas has transformed neurosurgical oncology, emphasizing the balance between maximizing tumor resection and preserving neurological functions (Gogos et al. 2020). While the practice has been extensively validated for tumors in the dominant hemisphere, its application to the non‐dominant hemisphere remains underexplored, despite growing evidence of its functional significance. Traditionally, the non‐dominant hemisphere, often associated with the right side in most individuals, was considered less critical for eloquent brain functions (Duffau 2018). This perception led to a preference for general anesthesia (GA) during surgeries for gliomas in these regions. However, recent studies challenge this notion, highlighting the critical roles of the non‐dominant hemisphere in visuospatial processing, social cognition, and emotional regulation (Bernard et al. 2018; Krall et al. 2015; Schuwerk et al. 2017). Evidence suggests that deficits in these domains, while subtler than language impairments, can profoundly impact patients' quality of life, particularly in their social and professional capacities (Gorgoraptis et al. 2019). Cognitive symptoms are frequent in patients with brain tumors, and they have a critical impact on complex activities of daily living, work, and functional independence (Parsons and Dietrich 2021). The complex interplay of multiple relapses of neurobehavioral dysfunction in patients with brain tumors implies that consistent monitoring represents an important part of comprehensive clinical care (Parsons and Dietrich 2021). The identification of alterations in neurocognitive function appears vital, since it may cause disruption of both local and distant brain networks, resulting in cognitive deficits (Parsons and Dietrich 2021; Tucha et al. 2000). Intraoperative brain mapping (ioBM) paradigms, initially developed to safeguard language functions in the dominant hemisphere, have begun to be adapted for the non‐dominant hemisphere. These techniques enable the real‐time assessment of cognitive functions such as spatial attention, social cognition, and non‐verbal communication. Analyzing the effect of treatment assessing neurobehavioral function of brain tumor patients (Scheibel et al. 1996; Maire et al. 1987) appears essential, since it provides supplementary information about cognitive functions, continuous feedback, and a dynamic view of brain functions (Skrap et al. 2016). By mapping cognitive domains intraoperatively, postoperative deficits may be minimized, thus achieving optimal resection outcomes. Despite these advances, the lack of standardized protocols and limited literature addressing non‐dominant hemisphere mapping pose significant challenges.

This narrative review aims to critically analyze the current literature data concerning the use of awake surgery for gliomas in the non‐dominant hemisphere, summarizing data on cognitive domains, neuropsychological assessment, mapping techniques, and cognitive and surgical outcomes.

## 2 Materials and Methods

A detailed search using the PubMed database was conducted to identify scientific articles published between 2015 and 2024 concerning ioBM paradigms and cognitive outcomes in patients with gliomas located in the right non‐dominant hemisphere (RH). This involved a combination of keywords and MeSH terms that captured key concepts such as awake surgery, brain mapping, gliomas, cognitive assessment, and non‐dominant hemisphere. The research aimed to retrieve studies that provided insights into the cognitive domains examined and the interaction between neuropsychological assessment, surgical techniques, and ioBM on cognitive outcomes in patients with non‐dominant hemisphere gliomas. The full search string used was:

((((“awake”[All Fields] OR “awakeness”[All Fields] OR “awakes”[All Fields] OR “awaking”[All Fields]) AND (“brain mapping”[MeSH Terms] OR (“brain”[All Fields] AND “mapping”[All Fields]) OR “brain mapping”[All Fields])) OR ((“intraop”[All Fields] OR “intraoperative”[All Fields] OR “intraoperatively”[All Fields]) AND (“mapped”[All Fields] OR “mapping”[All Fields] OR “mappings”[All Fields])) OR ((“awake”[All Fields] OR “awakeness”[All Fields] OR “awakes”[All Fields] OR “awaking”[All Fields]) AND (“surgery”[MeSH Subheading] OR “surgery”[All Fields] OR “surgical procedures, operative”[MeSH Terms] OR (“surgical”[All Fields] AND “procedures”[All Fields] AND “operative”[All Fields]) OR “operative surgical procedures”[All Fields] OR “general surgery”[MeSH Terms] OR (“general”[All Fields] AND “surgery”[All Fields]) OR “general surgery”[All Fields] OR “surgery s”[All Fields] OR “surgerys”[All Fields] OR “surgeries”[All Fields])) OR ((“brain”[MeSH Terms] OR “brain”[All Fields] OR “brains”[All Fields] OR “brain s”[All Fields]) AND (“surgery”[MeSH Subheading] OR “surgery”[All Fields] OR “surgical procedures, operative”[MeSH Terms] OR (“surgical”[All Fields] AND “procedures”[All Fields] AND “operative”[All Fields]) OR “operative surgical procedures”[All Fields] OR “general surgery”[MeSH Terms] OR (“general”[All Fields] AND “surgery”[All Fields]) OR “general surgery”[All Fields] OR “surgery s”[All Fields] OR “surgerys”[All Fields] OR “surgeries”[All Fields]))) AND (“non‐dominant”[All Fields] AND (“hemispheral”[All Fields] OR “hemisphere”[All Fields] OR “hemisphere s”[All Fields] OR “hemispheres”[All Fields] OR “hemispheric”[All Fields] OR “hemisphericity”[All Fields]) AND (“glioma”[MeSH Terms] OR “glioma”[All Fields] OR “gliomas”[All Fields] OR “glioma s”[All Fields]))) OR ((“right”[All Fields] OR “right s”[All Fields] OR “rightful”[All Fields] OR “rights”[All Fields]) AND (“hemispheral”[All Fields] OR “hemisphere”[All Fields] OR “hemisphere s”[All Fields] OR “hemispheres”[All Fields] OR “hemispheric”[All Fields] OR “hemisphericity”[All Fields]) AND (“glioma”[MeSH Terms] OR “glioma”[All Fields] OR “gliomas”[All Fields] OR “glioma s”[All Fields]))) AND (“cognition”[MeSH Terms] OR “cognition”[All Fields] OR (“cognitive”[All Fields] AND “functions”[All Fields]) OR “cognitive functions”[All Fields])

### 2.1 Eligibility Criteria

To ensure relevance and quality, studies were included that focused on patients undergoing awake surgery for gliomas in the non‐dominant hemisphere and reported neuropsychological assessment, tumor characteristics, extent of surgical resection, and cognitive outcomes. Articles published in English and subjected to peer review were considered eligible. Exclusions were applied to case reports, reviews, and studies that focused exclusively on dominant hemisphere gliomas. These criteria ensured that the studies analyzed were robust and directly related to the research question.

After these exclusion criteria, the search yielded 74 articles, which were screened meticulously. After removing duplicates, two independent reviewers (A.T. and G.S.) assessed the titles and abstracts for relevance. Full‐text articles of potential interest were examined in detail to confirm eligibility. Discrepancies in the selection process were resolved through consensus or consultation with a third reviewer (G.E.U.). Ultimately, 13 studies met the criteria and were included in the review.

## 3 Results

Most of the studies evaluated cognitive abilities before, during, and after awake neurosurgery. In particular, the following cognitive domains were explored: speech/motor language, visuospatial cognition, executive functions, social cognition, working memory, spatial attention, and sensory‐motor functions. Neuropsychological assessment (Table 2) was planned considering the anatomical location of the tumor, the neural network, but also the motivation of the patients and the social background (Table 1). Among the 13 papers considered, patients underwent intraoperative neuropsychological assessment using the “awake‐asleep‐awake” technique in most of the cases, and the “awake” technique in the other three cases. In the other studies, the technique used was not specified, while in all cases, ioBM included cortical and subcortical stimulation. Intraoperative neuropsychological assessment, the cognitive domain examined, and the mapping techniques used are summarized in Table 3. DES induced in a transient lesion producing performance deficits during the ioBM, allowing disclosure of many positive sites, although not on all occasions (Puglisi et al. 2019). The extent of resection (EoR) has been described by using qualitative terms (i.e., partial, subtotal, total, gross‐total, or supra‐total), quantitative volume values, or percentages in many studies (8/14). Studies that compared subjects who underwent surgery under GA and subjects who underwent awake surgery, a more extended tumor resection was registered (Rijnen et al. 2019; Prat‐Acín et al. 2021). Postoperative neuropsychological assessment was carried out in most cases between one and three months after surgery; in some studies, neuropsychological assessment was carried out after 6 months, and in one study after 18 months (Hartung et al. 2021).

**TABLE 1:** Demographic characteristics.

Author, year	Sample size	Number of RH glioma (awake surgery)	Average age	Age range	Female *n*	Right‐ handed *n*	Average education	Low Grade Glioma *n*	Average tumor volume (cm^3^)	Total resection %	Tumor site
Puglisi et al., 2019	63	63	43	16‐77	30	53	13,85	21	37,143	NI	F, F‐I, F‐T, F‐P, T‐I, F‐T‐P, F‐T‐I, P, T‐P, P‐O
Rijnen et al., 2019	38	27	41,7	NI	NI	NI	14,8	38	41,6	10,52	F
Prat‐Acìn et al., 2021	15	15	articu53	29‐69	8	NI	NI	15	NI	46,66	F,T,P,I
Hartung et al., 2021	22	22	41	21‐67	11	NI	NI	22	NI	NI	P
Herbet et al., 2015	49	16	35,53	18‐54	26	49	NI	49	NI	NI	DLPFC
Motomura et al., 2018	9	4	39,25	17‐49	2	3	NI	4	28,5	NI	SFG
Tomasino et al., 2023	29	14	44,5	NI	NI	10	14,74	10	NI	4,67	PF
Barberis et al., 2022	18	18	41,9	26‐58	6	0	NI	16,2	51	55	SFG, F, MFG, IFG
Charras et al., 2015	20	20	38,9	18‐65	15	18	14,1	20	NI	94,4	F, T, F‐T‐I, I, T‐I, T‐O, P
Nakajima et al., 2017	18	18	46,6	31‐68	10	NI	NI	18	NI	NI	SFG, SLF, cingulum
Nakajima et al., 2019	66	30	43	16‐73	27	NI	NI	NI	NI	NI	F, T, P, O
Nakajima et al., 2018	20	20	47,4	31‐68	NI	NI	NI	6	NI	75,7	F,P,T
Liu et al., 2020	90	41	47,6	31‐68	10	NI	NI	18	NI	NI	SFG, SLF, cingulum

Abbreviations: DLPFC, dorsolateral prefrontal cortex; F, frontal; F‐I, fronto‐insular; F‐P, fronto‐parietal; F‐T, fronto‐temporal; F‐T‐I, fronto‐temporo‐insular; F‐T‐P, fronto‐temporo‐parietal; I, insular; IFG, inferior frontal gyrus; MFG, medium frontal gyrus; NI, no information; O, occipital; P, parietal; PF, prefrontal; P‐O, parieto‐occipital; SFG, superior frontal gyrus; SLF, superior longitudinal fascicle; T‐I, temporo‐insular; T‐O, temporo‐occipital; T‐P, temporo‐parietal.

**TABLE 2:** Pre/post neuropsychological assessment.

Author, year	Cognitive functions examined	Neuropsychological tests
Puglisi et al., 2019	Language, praxis, attention, executive functions	Picture naming test, Rey figure, digit span, verbal and semantic fluency, Stroop test, trail making test, token test, attentional matrices, Progressive Rey Matrices,
Rijnen et al., 2019	Verbal memory, visual memory, motor speed, psychomotor speed, automatic response inhibition, cognitive flexibility, sustained attention, verbal skills, executive control	Verbal and semantic fluency, Stroop test, verbal memory test, visual memory test, finger tapping test, shifting attention test, continuous performance test
Prat‐Acìn et al., 2021	Naming, nonverbal semantic associations, multitasking, Working Memory, attention, visuospatial cognition, Theory of Mind, facenaming process	Line bisection test, dual task, reading the mind in the eyes test, DO80, semantic fluency, palm and pyramids tree test, famous faces test
Hartung et al., 2021	Cognitive flexibility	Stroop test, trail making test
Herbet et al., 2015	Speech/motor language, semantic associations	DO80, pyramids and palm trees test
Motomura et al., 2018	Language, executive function	Picture naming test, standard language test for Aphasia, WAIS‐III, WMS‐R, frontal assessment battery
Tomasino et al., 2023	Manipulation, abstract reasoning, cognitive flexibility, psychomotor speed, selective attention, attentional shifting, strategic reasoning, response plausibility, automatic response inhibition, motor speed, visuoperceptive functions, planning, WM, short‐term memory, visuospatial cognition	Line bisection test, Rey figure, clock drawing test, digit span, trail making test, progressive rey matrices, oldfield, cognitive estimates, metaphors and idioms comprehension, copy of figures
Barberis et al., 2022	Executive functions, cognitive flexibility, Planning, inhibition, attention, short‐term memory, Spatial cognition, processing speed, social cognition, semantics, conceptualization, anosognosia, amimia	Rey figure, Target cancellation test, DO80, Digit Span, Verbal and semantic fluency, Stroop test, Trail Making Test
Charras et al., 2015	Attention, executive functions, social cognition	Line bisection test, cancellation test, reading the mind in the eyes test, Ekman's faces
Nakajima et al., 2017	Social cognition, visuospatial cognition, WM	N‐back test, expression recognition test, picture arrangement task, line bisection test, target cancellation test, verbal and semantic fluency
Nakajima et al., 2019	Working memory, theory of mind, visuospatial cognition, language	N‐back test, expression recognition test, picture arrangement task, line bisection test, picture naming test, Rey figure, clock drawing test, cancellation test, KPS scale,
Nakajima et al., 2018	High‐level mentalizing	Picture arrangement task
Liu et al., 2020	NI	NI

Abbreviations: NI, no information; WAIS‐III Wechsler adult intelligence scale; WMS‐R, Wechsler memory scale‐revised, WM working memory.

**TABLE 3:** Intraoperative brain mapping technique, cognitive domains examined, and intraoperative neuropsychological assessment.

Author, year	Intraoperative technique	Mapping cognitive domain examined	Neuropsychological tests
Puglisi et al., 2019	NS	Executive functions	Stroop test
Rijnen et al., 2019	Awake	NI	NI
Prat‐Acìn et al., 2021	Asleep‐awake‐asleep	Visuospatial cognition, social cognition, executive functions, memory	Line Bisection task, dual task, reading the mind in the eyes test, short‐term memory, number counting (1‐10), DO80, famous faces test
Hartung et al., 2021	NS	NS	NS
Herbet et al., 2015	NI	NI	NI
Motomura et al., 2018	Asleep‐awake‐asleep	Speech‐motor language, visuospatial cognition, memory	Picture naming test, N‐back test, line bisection test, counting task, digit span
Tomasino et al., 2023	Awake	Executive functions, speech/motor language, memory, motor	Stroop test, trail making test, short‐term memory test, working memory test, symbol digit modalities test, metaphor comprehension
Barberis et al., 2022	Awake	Sensorimotor function, social cognition, Speech/motor language, executive functions	Limb movement, dual task, reading the mind in the eyes test, PPTT,
Charras et al., 2015	NS	NS	NS
Nakajima et al., 2017	Asleep‐awake‐asleep	Visuospatial cognition, Social cognition, Executive functions	N‐back test, line bisection test, expression recognition test, dual task, Stroop test
Nakajima et al., 2019	Asleep‐awake‐asleep	Speech/motor language, social cognition, praxis	Picture naming test, N‐back test, line bisection test, expression recognition test, picture arrangement task,
Nakajima et al., 2018	Asleep‐awake‐asleep	Sensorimotor functions, language, WM, visuospatial cognition, social cognition	Limb movement, sensorial perception, picture naming, N‐back test, line bisection test, expression recognition test, picture arrangement task
Liu et al., 2020	Asleep‐awake‐asleep	Social cognition, visuospatial cognition, WM, Speech/motor language	Picture naming test, N‐back test, line bisection test, counting task, PPTT, theory of mind test

Abbreviations: DO80, oral image naming test; NI, no information; NS, not specified; PPTT, pyramids and palm trees test; WM, working memory.

### 3.1 Speech/Motor Language

Speech/motor language was evaluated by 8 out of the 13 studies. The neuropsychological assessment included the pyramids and palm tree test (PPTT) (Prat‐Acín et al. 2021; Herbet et al. 2015), the standard language test for Aphasia (Motomura et al. 2018), metaphors and idioms comprehension (Tomasino et al. 2023), the Orale Image Naming test (DO80) (Prat‐Acín et al. 2021; Herbet et al. 2015; Barberis et al. 2022), the Token test (Puglisi et al. 2019), and verbal and semantic fluency (Puglisi et al. 2019; Rijnen et al. 2019; Prat‐Acín et al. 2021; Barberis et al. 2022; Charras et al. 2015; Nakajima et al. 2017). At the pre‐operative assessment, mild or moderate cognitive deficits in semantic cognition were detected (Barberis et al. 2022). In another case, no speech/motor language deficits were found (Herbet et al. 2015). For the ioBM, the picture naming test was used in four cases (Motomura et al. 2018; Nakajima et al. 2019; Nakajima et al. 2018; Liu et al. 2020), while the pyramids and palm trees test for nonverbal semantics was used in three cases (Herbet et al. 2015; Barberis et al. 2022; Liu et al. 2020) and the DO80 in two cases (Prat‐Acín et al. 2021; Herbet et al. 2015). The metaphor comprehension test was used just in one case (Tomasino et al. 2023). DES in the frontal region provoked articulatory disorders, anomia, and semantic paraphasia (Prat‐Acín et al. 2021). Prat‐Acìn and colleagues (Prat‐Acín et al. 2021) observed speech arrest in most of their patients during frontal and insular stimulation, while Motomura and collaborators (Motomura et al. 2018) found language interferences inducing semantic paraphasias and articulatory disorders in patients during stimulation of IFOF and FAT. Furthermore, a role of the right frontal lobe in emotional prosody was found (Tomasino et al. 2023). After surgery, no decrease in metaphor comprehension was found (Tomasino et al. 2023), while other patients showed persistent deficits in this cognitive function (Barberis et al. 2022).

### 3.2 Attention and Visuospatial Cognition

Since they were considered by 9 of the 13 studies, attention and visuospatial cognition were two of the most assessed cognitive functions. The neuropsychological assessment included the line bisection test (Prat‐Acín et al. 2021; Tomasino et al. 2023; Charras et al. 2015; Nakajima et al. 2017; Nakajima et al. 2019), the target cancellation test (Charras et al. 2015; Nakajima et al. 2019), the trail making test (Puglisi et al. 2019; Hartung et al. 2021; Tomasino et al. 2023; Barberis et al. 2022), the continuous performance test (Rijnen et al. 2019), the digit symbol substitution test (Tomasino et al. 2023), the shifting attention test (Rijnen et al. 2019), and the attentional matrices (Puglisi et al. 2019). In some cases, the pre‐operative neuropsychological assessment showed no frank spatial or visual exploration deficit (Charras et al. 2015; Nakajima et al. 2017), while Barberis and colleagues (Barberis et al. 2022) found distractibility in 30% of cases. The line bisection test was used in 6 of 13 of the studies considered (Prat‐Acín et al. 2021; Motomura et al. 2018; Nakajima et al. 2017; Nakajima et al. 2019; Nakajima et al. 2018; Liu et al. 2020), and it was the most used ioBM neuropsychological test. Some studies found that DES of the supramarginal gyrus and the second branch of the superior longitudinal fascicle (SLF) produced disturbances of spatial cognition with right deviations and disruption of the vestibular inputs (Charras et al. 2015; Nakajima et al. 2019). Moreover, a role of the medial superior and middle frontal gyri in low visuospatial cognitive accuracy was found (Tomasino et al. 2023). At the follow‐up, persistent visuospatial cognition deficits were found (Nakajima et al. 2017; Nakajima et al. 2019), while Barberis and colleagues (Barberis et al. 2022), and Tomasino and colleagues (Tomasino et al. 2023) found decreased performance also in attention and spatial cognition with a decrease in performance at the line bisection test. In one case, despite the significance of cognitive data reported after surgery, a decrease in visuospatial cognitive accuracy was detected (Tomasino et al. 2023).

### 3.3 Executive Functions

Executive functions were analyzed in 8 of the 13 studies. The neuropsychological assessment included the clock drawing test (Tomasino et al. 2023; Nakajima et al. 2019), the frontal assessment battery (Motomura et al. 2018), the Stroop test (Puglisi et al. 2019; Rijnen et al. 2019; Hartung et al. 2021; Barberis et al. 2022; Charras et al. 2015), and cognitive estimations (Tomasino et al. 2023). In general, patients performed well at the pre‐surgery assessment, and deficits involved more cognitive estimation and inhibition abilities (Rijnen et al. 2019; Tomasino et al. 2023). Barberis and colleagues (Barberis et al. 2022) reported mild executive function deficit concerning speed processing in 50% of cases, irritability in 15%, and fatigability in 20%. Intraoperatively, DES revealed that frontal lobe lesions were associated with interference and cognitive control deficits with the involvement of the inferior frontal gyrus (IFG), dorsolateral prefrontal cortex (DLPFC), anterior cingulate, and pre‐supplementary motor area (pre‐SMA) (Puglisi et al. 2019). At the same time, parietal lesions lead to alteration of cognitive flexibility (Hartung et al. 2021). For the ioBM of executive functions the Stroop Test, and the dual task were adopted frequently (Puglisi et al. 2019; Tomasino et al. 2023; Nakajima et al. 2017), while the trail making test was adopted only one time by Tomasino and colleagues (Tomasino et al. 2023). It has enlightened the role of the right FAT in executive functions, encompassing inhibition, planning, monitoring, and cognitive flexibility (Rijnen et al. 2019; Hartung et al. 2021). Moreover, an involvement of the right parietal lobe in the integration of information was found (Hartung et al. 2021). After surgery, a decrease in speed of processing and in multitasking was found (Barberis et al. 2022). Less commonly, reduced speed processing, lack of motivation, irritability, mood disorders, and difficulties with time management were detected (Barberis et al. 2022). No improvement in TMT performance in patients with smaller resection volumes and not in the larger resection volumes was found (Hartung et al. 2021). A slight decrease in the performance of the clock drawing test and cognitive estimation was registered by Tomasino and colleagues (Tomasino et al. 2023), while a significant deterioration of cognitive flexibility was observed by Rijnen and colleagues (Rijnen et al. 2019). By contrast, an improvement in cognitive control was found by Puglisi and colleagues (Puglisi et al. 2019).

### 3.4 Social Cognition

Social cognition was investigated in 5 of the 13 studies. The neuropsychological assessment included the reading the mind in the eyes test (Prat‐Acín et al. 2021) and the expression recognition test (Nakajima et al. 2017; Nakajima et al. 2019). For the ioBM, the expression recognition test was used in three cases (Nakajima et al. 2017; Nakajima et al. 2019; Nakajima et al. 2018), while a revised version of the reading the mind in the eyes test was used in two cases (Prat‐Acín et al. 2021; Barberis et al. 2022), as well as the picture arrangement test (Nakajima et al. 2019; Nakajima et al. 2018). The theory of mind test was used only in one case (Liu et al. 2020). A role of the frontal lobe in emotion recognition was reported (Herbet et al. 2015; Nakajima et al. 2018), and it is made possible by parallel functioning of two subsystems in the RH: the first mediated by the right arcuate fascicle/SLF complex, which subserve perceptual aspects and emotional empathy, the second by the right cingulum, which supports cognitive empathy (Herbet et al. 2015). It has also been shown that preserving both the dorsal inferior fronto‐occipital fasciculus (IFOF) and the uncinate fasciculus (UF) is crucial for keeping mentalizing abilities intact (Nakajima et al. 2018), and that a disconnection of the UF predicted low empathy (Herbet et al. 2015). Concerning neuropsychological outcome, a persistent deficit in the social cognition domain after surgery was detected in patients with smaller resection volumes (Barberis et al. 2022), and a worsening in mentalizing tasks in one patient was detected by Prat‐Acìn and colleagues (Prat‐Acín et al. 2021). By contrast, Nakajima and colleagues (Nakajima et al. 2018) found that patients who underwent ioBM presented the same score in high‐level mentalizing at 3 months after surgery.

### 3.5 Short‐Term Memory and Working Memory

Short‐term memory and WM were assessed in 6 of the 13 studies. The neuropsychological assessment included the digit span, both forward and backward (Puglisi et al. 2019; Tomasino et al. 2023; Barberis et al. 2022), verbal and visual memory test (Rijnen et al. 2019). Preoperatively, Barberis and colleagues (Barberis et al. 2022) found a mild compromise of verbal short‐term memory in 45% of cases. Intraoperatively, a robust assessment for short‐term memory and WM, by using digit span, both backward and forward, visual and spatial N‐back task, was proposed by different authors (Motomura et al. 2018; Nakajima et al. 2019; Liu et al. 2020). Other measurements proposed were the counting task (Motomura et al. 2018; Liu et al. 2020), the short‐term memory test (Prat‐Acín et al. 2021; Tomasino et al. 2023), the famous faces test (Prat‐Acín et al. 2021), and the working memory test (Tomasino et al. 2023). A disruption of verbal and spatial WM was registered during the stimulation of DLPFC (Motomura et al. 2018). After surgery, Motomura and colleagues (Motomura et al. 2018) also found verbal and spatial WM functions intact, while disruption of verbal short‐term memory was found by Barberis and colleagues (Barberis et al. 2022).

### 3.6 Sensorimotor Function

Only 2 of the 13 studies were taken into consideration. Preoperatively, tests for sensorimotor function, such as the finger tapping test (Rijnen et al. 2019) and the dual task (Prat‐Acín et al. 2021) were used. Interestingly, it has been found that DES of the RH allowed to find positive motor sites involved mainly in the face, or mouth area (Tomasino et al. 2023), and a role of bimanual coordination tasks was documented (Barberis et al. 2022). After surgery, a loss of bimanual coordination was found in just one case (Barberis et al. 2022).

## 4 Discussion

To date, most studies have evaluated the potential of awake surgery for glioma removal in the left hemisphere, given its role in language production and comprehension. Yet it is known that the crucial role of RH in many brain functions, such as in movement execution and control, visual processes, spatial cognition, language, and nonverbal semantic processing, executive functions, and emotional processes, challenges the idea of the existence of a non‐dominant hemisphere. This was also confirmed by previous studies (Mamadaliev et al. 2024; Vilasboas et al. 2017). These findings cast doubt also on conventional surgical methods and point to a change in perspective regarding how the brain works and how surgery affects patient outcomes. However, only a few studies on this topic are available. Therefore, the goal of the present review was to contribute to defining the current state of the art concerning intraoperative neuropsychological assessment for the RH hemisphere. In order to do so, 13 papers were taken into consideration, and the results point to some clinical ramifications.

The most frequently assessed cognitive functions were attention and visuospatial cognition, executive functions, and speech/motor language. Social cognition, short‐term memory and WM, and sensorimotor functions were also assessed, but less frequently.

Some studies found that DES of the supramarginal gyrus and the second branch of the SLF produces disturbances of spatial cognition with right deviations and disruption of the vestibular inputs, suggesting a role of the right corticosubcortical frontoparietal network in spatial awareness, visuospatial attention, and cognition (Charras et al. 2015; Nakajima et al. 2019), but also in visual scene processing, as found in a previous study (Vilasboas et al. 2017). Indeed, since DES may generate a breakdown in conscious experience, a role of the RH in maintaining arousal and the consciousness of the external environment is plausible (Vilasboas et al. 2017). Low visuospatial accuracy was associated with disruption of the medial superior and middle frontal gyri, suggesting the involvement of the right hemisphere in spatially oriented actions such as object manipulation, navigation, and visual‐motor coordination. These functions are essential for a wide range of daily activities, including driving, navigating through physical environments, and performing professional tasks that require spatial precision. Consequently, preserving these areas during surgery is critical to maintaining the patient's autonomy and functional independence (Charras et al. 2015). Thus, this function seems to be supported by ventral and dorsal attention networks with other attentional functions such as arousal and vigilance, saliency detection, and reorienting of attention (Charras et al. 2015), and frequent testing seems necessary to prevent deficits. RH is involved also in executive functions, which include cognitive flexibility, cognitive control, inhibition, and planning and monitoring (Rijnen et al. 2019; Tomasino et al. 2023): ioBM appears vital in preventing the deterioration of these abilities, which may have a strong negative impact on individuals’ quality of life, since it results in social exclusion and limited professional development (Hartung et al. 2021). Moreover, the role of ioBM in the preservation of emotional prosody was also found (Tomasino et al. 2023). Intraoperative DES combined with preoperative and postoperative assessment brought new evidence to support the idea of a crucial role of the RH in mentalizing (Nakajima et al. 2019; Nakajima et al. 2018), and emotional processing (Tomasino et al. 2023). That is, it has been shown that ioBM contributes to the maintenance of high‐level mentalizing at 3 months after surgery (Nakajima et al. 2018). Also, ioBM enlightened the role of the right FAT in WM (Nakajima et al. 2019), and of the DLPFC for verbal and spatial WM (Motomura et al. 2018), allowing for preservation of these functions after surgery (Motomura et al. 2018). However, this is not confirmed by other studies (Barberis et al. 2022). Lastly, sensorimotor functions have been poorly investigated, however, it is interesting to note that RH has a role in bimanual coordination, and ioBM seems to protect this ability in most cases (Barberis et al. 2022). Taking these results into consideration, ioBM in conjunction with awake surgery has been shown to be a successful method for optimizing tumor excision while maintaining vital functions. According to the research evaluated, patients who had awake surgery had better long‐term survival rates and larger EOR than those treated under GA. With its capacity to increase EOR while maintaining cognitive abilities, awake surgery using ioBM ought to be regarded as a common treatment for non‐dominant hemisphere gliomas. Future studies should concentrate on the following areas:

1. Creating a single set of cognitive tests for evaluating right hemisphere function during surgery.
2. Establishing long‐term research projects to track cognitive recovery.
3. Looking at how modern imaging methods, such tractography, can be used to direct surgery and enhance functional results.
4. Cooperation within disciplines to guarantee reliable data collection and standardized techniques. It is worth noting that the inconsistent methods and cognitive evaluation procedures used in different studies are significant issues that this analysis highlights. Although most of the included studies evaluated cognitive abilities before, during, and after surgery, there was considerable variation in the assessments used. These discrepancies emphasize the necessity for uniform testing procedures and make it challenging to compare findings across studies. Moreover, the wide range of assessment instruments reflects the RH's intricate functional repertoire. For instance, according to the reviewed research (Herbet et al. 2015; Charras et al. 2015; Nakajima et al. 2019), visuospatial and social cognition depend on pathways such as the SLF and IFOF. However, it was difficult to completely comprehend the functional importance of these networks due to variations in testing methodologies. This emphasizes how crucial it is to provide focused evaluation techniques for non‐dominant hemisphere functions.

In addition, the potential to fully benefit from awake surgery is limited by the absence of standardized mapping techniques for the right hemisphere. To develop useful and trustworthy intraoperative activities suited to the unique capabilities of the right hemisphere, more study is required.

Variability in reporting postoperative cognitive outcomes is another of the examined studies' limitations. Some studies only gave a limited amount of information on recovery trajectories, whereas others provided comprehensive longitudinal data. The findings' potential to be applied broadly is limited by this discrepancy, small sample sizes, and diverse tumor sites. Furthermore, some research's applicability to more general inquiries regarding right hemisphere gliomas is limited by their lobe‐specific focus.

And more, the heterogeneity in cognitive assessment tools across studies hinders direct comparisons and meta‐analytical approaches. Future research should focus on establishing standardized neuropsychological testing protocols for intraoperative and postoperative evaluations. In addition, longitudinal studies tracking cognitive recovery over extended periods would provide valuable insights into the long‐term impact of awake surgery on high‐order cognitive functions. Future trials should also explore the effectiveness of personalized rehabilitation strategies in mitigating postoperative deficits. Incorporating multimodal neuroimaging techniques, such as functional magnetic resonance imaging (fMRI) and diffusion tensor imaging (DTI), may further elucidate the neural networks underlying cognitive recovery and help refine surgical mapping techniques.

Indeed, recent advancements in artificial intelligence (AI), particularly machine learning (ML), offer new possibilities for enhancing both preoperative planning and intraoperative functional preservation. For instance, support vector machines (SVMs) and convolutional neural networks (CNNs) have been applied to preoperative neuroimaging data (e.g., fMRI and DTI) to classify brain regions based on their likelihood of being functionally eloquent. These algorithms help predict post‐surgical cognitive outcomes by learning patterns from large datasets of imaging and neuropsychological performance. Mrah et al. demonstrated how such predictive models can guide surgical strategies by identifying resections that put patients at risk of long‐lasting deficits in domains like set‐shifting, thus informing whether specific intraoperative tasks should be included in the mapping protocol (Mrah et al. 2022).

It is essential to acknowledge that many right‐hemisphere cognitive deficits observed in the immediate postoperative period—such as impairments in emotion recognition and cognitive flexibility—tend to resolve over time in most patients. This recovery trajectory has been detailed in the review by Herbet et al. (2024). As a result, there is a potential risk of prematurely halting tumor resection based on transient functions, thereby compromising the oncological benefit. To address this issue, Mandonnet et al. have proposed a structured process for introducing new cognitive tasks into intraoperative mapping protocols (Mandonnet et al. 2020). This involves clearly identifying which cognitive functions are associated with persistent deficits and determining in which patient profiles testing should be prioritized. Moreover, the integration of predictive models through AI and machine learning could serve as a valuable tool to stratify patients according to their recovery potential and guide surgical decision‐making accordingly.

Tractography‐guided neurosurgical planning has already improved the preservation of key white matter pathways (Essayed et al. 2017), but further advancements in AI‐assisted brain mapping could enhance real‐time intraoperative decision‐making (Mut et al. 2024). Future research should explore how these technologies can be integrated into clinical workflows to optimize both oncological and functional outcomes in patients undergoing awake surgery for right hemisphere gliomas.

## 5 Conclusions

This paper represents an attempt to contribute to defining a standardized protocol for intraoperative assessment of cognitive functions in the non‐dominant hemisphere. It has been reported that cognitive and behavioral deficits after brain surgery are frequent also in the right hemisphere. The intraoperative monitoring of functions like visuospatial cognition, executive functions, and social cognition seems to have a crucial role in preventing the deterioration of abilities that can significantly impact a patient's quality of life. Patients with low‐grade gliomas have an extended survival; therefore, to guarantee the best possible quality of life for them appears necessary. Consequently, the resection of right‐sided tumors performed using awake surgery with cortical and axonal electrostimulation paradigms appears to be promising solution.

## Author Contributions

**Alice Tomaselli**: methodology, conceptualization, investigation, validation, formal analysis, writing – original draft, writing – review and editing. **Antonina Luca**: conceptualization, methodology, supervision, writing – review and editing, project administration, resources. **Gianluca Ferini**: validation, formal analysis, visualization, writing – review and editing. **Giuseppe Emmanuele Umana**: conceptualization, supervision, funding acquisition, project administration, resources, writing – review and editing. **Bipin Chaurasia**: methodology, software, data curation, investigation, visualization, writing – review and editing. **Gianluca Scalia**: conceptualization, methodology, software, data curation, investigation, validation, supervision, visualization, project administration, writing – original draft, writing – review and editing.

## Ethics Statement

The authors have nothing to report.

## Consent

The authors have nothing to report.

## Conflicts of Interest

The authors declare no conflicts of interest.

### Peer Review

The peer review history for this article is available at https://publons.com/publon/10.1002/brb3.70604.

## Data Availability

No datasets were generated or analyzed during the current study.
